# Adaptation and Immunity

**DOI:** 10.1371/journal.pbio.0020307

**Published:** 2004-09-14

**Authors:** Eddie C Holmes

## Abstract

The ongoing battle between hosts and pathogens has long been of interest to evolutionary biologists.

The ongoing battle between hosts and pathogens has long been of interest to evolutionary biologists. Because hosts and pathogens act as environments for each other, their intertwined struggle for existence is both continual and rapid. At the molecular level, this cycle of environmental change and evolutionary response means that mutations are continually being tried out by natural selection. It is therefore little wonder that the host and pathogen genes that control infection and immunity frequently show high levels of genetic diversity and present some of the best examples of positive selection (adaptive evolution) reported to date (Yang and Bielawski 2000). In particular, rates of nonsynonymous substitution per site (resulting in an amino acid change; d_N_) often greatly exceed those of synonymous substitution per site (silent change; d_S_), as expected if most mutations are fixed because they increase fitness (Figure 1).

**Figure 1:**
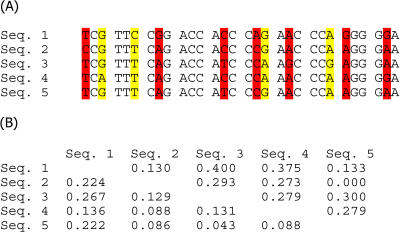
Measuring Selection Pressures by Comparing the Ratio of Nonsynonymous to Synonymous Substitutions Per Site (A) Classification of substitutions. Nonsynonymous substitutions (red) are those that change the amino acid sequence of the protein encoded by the gene, while the degeneracy of the genetic code ensures that synonymous substitutions (yellow) result in the same amino acid sequence. (B) Calculation of d_N_/d_S_. By assuming that synonymous mutations are neutral and fixed by random genetic drift, it is possible to determine the mode of selection acting on nonsynonymous mutations. If all nonsynonymous substitutions were neutral, then their rate of occurrence per site (d_N_) would be the same as that of synonymous substitutions per site (d_S_), so that d_N_/d_S_ equals one. A lower ratio of nonsynonymous to synonymous substitutions per site (d_N_/d_S_ < 1) means that some proportion of the nonsynonymous mutations are deleterious and removed by purifying selection. Conversely, positive selection fixes advantageous nonsynonymous mutations faster than genetic drift fixes synonymous mutations (d_N_/d_S_ > 1), although this is usually restricted to a small proportion of amino acid sites within any gene. In the hypothetical example of five gene sequences shown here, with d_S_ given above the diagonal and d_N_ below the diagonal, there is no evidence for positive selection because mean d_N_/d_S_ the (0.577) is less than one.

At the host level, most studies of the selection pressures acting on immune system genes have concentrated on genes implicated in the adaptive immune response against microbial pathogens, particularly those producing antibodies (Sitnikova and Nei 1998; Sumiyama et al. 2002), or genes encoding reconnaissance molecules known as the major histocompatibility complex (MHC), which control the action of T-cells (Hughes and Nei 1988; Yeager and Hughes 1999) (Box 1). As its name suggests, the role of the adaptive immune response is to stimulate and ‘memorise’ immunity to specific pathogens. As microbial pathogens such as viruses are both abundant and rapidly evolving, positive selection on components of the adaptive immune response is often very strong (Yeager and Hughes 1999). Far less attention has been directed toward the less specific innate (‘nonadaptive’) immune response, even though this response requires a wide array of genes and acts as the front line of immune defence (Box 1). Would we expect the same strength of positive selection on a generalized pathogen control system? This is a question of fundamental importance because the luxury of adaptive immunity is not available to most organisms, having probably evolved along with the vertebrates (Bartl et al. 1994), whereas the more widespread innate immune system is often depicted as a primitive characteristic.

## Molecular Evolution of the Innate Immune System

The genes involved in innate immunity have recently come under the molecular evolutionists' gaze. One important group are the defensins, a large class of short antimicrobial peptides that constitute an effective immune response team in organisms as diverse as plants and primates (Boman 1995). Because defensins are cationic (positively charged), they are able to interact with negatively charged molecules on the surface of microbes and permeate their membranes. Sequence analyses of defensins and similar antimicrobial peptides have revealed the telltale signatures of positive selection, with d_N_ greater than d_S_ in many comparisons (Hughes 1999; Duda et al. 2002; Maxwell et al. 2003). Other genes of the innate immune system also seem to be subject to powerful positive selection. One dramatic example described in this issue of *PLoS Biology* is the APOBEC3G gene of primates (Sawyer et al. 2004). This case is especially striking because rather than killing pathogens through protein or cellular interactions, like most immune genes, APOBEC3G works by manipulating the genome sequence of the invading microbe.

The genomes of primates contain a family of nine APOBEC genes that encode enzymes involved in the editing of RNA and/or DNA through the deamination of cytosine (C), so that this nucleotide mutates to uracil (U). This is essential for various aspects of cellular function. APOBEC1, for example, is involved in the C→U editing of apolipoprotein B mRNA (therein christening the family), while another family member, the activation-induced deaminase, has a vital role in adaptive immunity in that it assists in the diversification of antibodies. Two more enzymes, APOBEC3G and APOBEC3F, form part of the innate immune system; they function as antiviral agents and are being intensively studied in the context of infection with the human immunodeficiency virus (HIV), the cause of AIDS. In particular, APOBEC3G targets the reverse transcription step of the HIV life cycle, in which the viral genomic RNA is converted into proviral DNA, which is then integrated into the host genome (Mangeat et al. 2003). APOBEC3G-induced deamination at this stage results in monotonous guanine-to-adenine (G→A) nucleotide changes, a phenomenon called G→A hypermutation that had long been noted by HIV researchers without a clear understanding of its cause. We now know that G→A hypermutation is part of the innate immune response to retroviral infections.

Although there is still some debate over exactly how APOBEC3G leads to viral eradication, the most likely scenario is that G3A hypermutation results in the generation and incorporation of a multitude of deleterious mutations that fatally disrupt viral functions. This strategy is likely to work well for retroviruses like HIV because their genomes are so compact that individual sequence regions often perform multiple functions. Under these cramped conditions, most mutations are likely to severely disrupt some aspect of viral function and thereby reduce fitness (Holmes 2003). Indeed, it has been estimated that the deleterious mutation rate in viruses that replicate using RNA polymerases (either reverse transcriptase in the case of retroviruses or RNA-dependent RNA polymerase for other RNA viruses) is on the order of one error per replication cycle, so that many of the viral progeny produced by replication are defective (Elena and Moya 1999). HIV is normally able to overcome this burden of deleterious mutation because of its remarkable reproductive power; each day, on the order of 10^10^ virions are produced in a single infected individual (Perelson et al. 1996), so that enough fit and able recruits will make it through to the next generation.

## Treating RNA Virus Infections Through Lethal Mutagenesis

**Figure 2:**
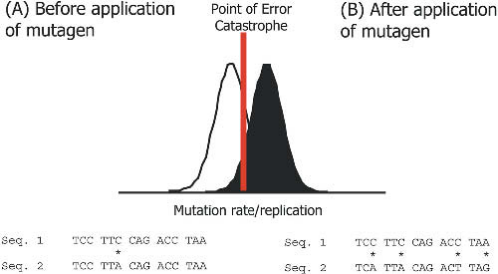
Lethal Mutagenesis As a Means of Controlling RNA Virus Infections (A) In a viral population prior to the application of mutagens, the mean error rate (white) is on the order of one per genome per replication (mutations marked by asterisks). (B) If a mutagen such as ribavirin is then applied to an infected patient, the mean error rate of the virus (black) is increased so that the population crosses a threshold of ‘error catastrophe’; after this point fitness declines dramatically and the population crashes. This drug-induced lethal mutagenesis seems to work more efficiently when it is used in combination with drugs that reduce the rate of viral replication.

The high mutation rates of RNA viruses mean that adaptively useful genetic variation is produced frequently. The rub, however, is that fitness-enhancing mutations are a small minority, and the preponderance of deleterious mutations means that RNA viruses live on the edge of survival (Domingo 2000). By increasing the rate at which deleterious mutations appear, APOBEC3G pushes viruses over this edge, causing a form of ‘lethal mutagenesis’ that results in their destruction; the rate of mutation becomes so high that no genome can reproduce itself faithfully, and the population crashes. Intriguingly, researchers designing new antiviral drugs have also begun to realise that forcing viruses into this sort of ‘error catastrophe’ might be an effective way to treat them (Figure 2). There are a growing number of studies in which mutagens, such as ribavirin and 5-fluorouracil, are applied to viral infections in vitro and in vivo, including HIV, in the hope that these will induce so many deleterious mutations that the virus suffers an error catastrophe and is cleared (Loeb et al. 1999; Sierra et al. 2000; Crotty et al. 2001; Ruiz-Jarabo et al. 2003). The results produced to date are highly encouraging, particularly when these error-inducing drugs are combined with more conventional treatment strategies that aim to reduce the rate of viral replication (Pariente et al. 2001). The discovery that a natural antiviral agent, APOBEC3G, probably works in much the same way should provide even more encouragement.

Sadly, however, the pathogens have fought back. The anti-HIV properties of APOBEC3G were discovered because most viral strains escape its neutralising properties. Lentiviruses like HIV possess a gene that encodes an protein called Vif (‘viral infectivity factor’) that counters APOBEC3G (Sheehy et al. 2002). Hence, it is probably only in naturally occurring Vif-defective mutants that APOBEC3G is effective against HIV. Furthermore, because positive selection on APOBEC3G has operated for at least 30 million years and lentiviruses in general, and HIV in particular, are likely to be more recently evolved than this, it is clear that a broad range of retroviral pathogens have been responsible for the adaptive evolution of this particular immune gene (Sawyer et al. 2004). Given the frequency with which the remnants of past retroviral infections are found in the mammalian genome (Smit 1999), in the form of usually defunct endogenous retroviruses (Box 1), it is likely that our genomes are continually bombarded with retroviruses like HIV but that the majority are cleared by innate immune mechanisms like APOBEC3G. It is possible that the retroviruses that successfully infect us are those, like HIV, that have managed to evolve strategies to avoid the destructive capacities of APOBEC3G.

The intense selective pressure on the defensins and APOBEC3G illustrates that although the innate immune response is generalist in its action, it is as highly and intricately evolved as its better-studied ally, the adaptive immune system. Rather than being an evolutionary remnant, it is a dynamic and continually adapting system. Less clear is whether other host proteins act in the same manner as APOBEC3G. In particular, the most common and destructive pathogens faced by humans and other mammals are RNA viruses, such as influenza A, yellow fever, and hepatitis C. In most cases, our ability to survive these viral infections is simply a combination of good luck and good breeding; with the right combination of MHC alleles, itself a function of population history and what we by chance inherit from our parents, some individuals may be more able to fight off viral infections than others. The ubiquity of RNA viruses hints that our genomes might also contain an innate, yet highly adapted, defence system that targets this abundant class of pathogens by manipulating their mutation rate. Although utilizing lethal mutagenesis might one day be an important way to design new drugs against a variety of viral pathogens, it would come as no surprise if nature got there first.

**Box 1. Glossary**

**Adaptive immune system.** The pathogen-specific part of the vertebrate immune system. It is comprised of two major arms, antibodies (the humoral response) and T-cells (the cellular response), both of which lay down an immunological memory for future defence.

**Innate immune system.** The nonspecific part of the vertebrate immune system. It has a wide variety of components, ranging from lysozymes in saliva to cytokines, defensins, interferons, and natural killer cells in a variety of tissues.

**Endogenous retroviruses.** The (usually) dead remnants of functional retroviruses that are now passed on through the germ line like normal genes. It has been estimated that approximately 5% of the human genome is composed of endogenous retroviruses.

## Abbreviations

C: cytosine
d_N_: rate of nonsynonymous substitution per site
d_S_: rate of synonymous substitution per site
G→A: guanine-to-adenine
HIV: human immunodeficiency virus
MHC: major histocompatibility complex
U: uracil
